# Exploratory behavior of re-orienting foragers differs from other flight patterns of honeybees

**DOI:** 10.1371/journal.pone.0202171

**Published:** 2018-08-29

**Authors:** Jacqueline Degen, Thomas Hovestadt, Mona Storms, Randolf Menzel

**Affiliations:** 1 Department of Biology, Freie Universität Berlin, Berlin, Germany; 2 Department of Animal Ecology and Tropical Biology, University of Würzburg, Würzburg, Germany; Philipps-Universitat Marburg Fachbereich Biologie, GERMANY

## Abstract

Honeybees, *Apis mellifera*, perform re-orientation flights to learn about the new surroundings of the hive when their hive is transported to a new location. Since the pattern of re-orientation flights has not yet been studied, we asked whether this form of exploratory behavior differs from the well described exploratory orientation flights performed by young honeybees before they start foraging. We also investigated whether the exploratory components of re-orientation flights differ from foraging flights and if so how. We recorded re-orientation flights using harmonic radar technology and compared the patterns and flight parameters of these flights with the first exploratory orientation flights of young honeybees and foraging flights of experienced foragers. Just as exploratory orientation flights of young honeybees, re-orientation flights can be classified into short- and long-range flights, and most short-range re-orientation flights were performed under unfavorable weather conditions. This indicates that bees adapt the flight pattern of their re-orientation and orientation flights to changing weather conditions in a similar way. Unlike exploratory orientation flights, more than one sector of the landscape was explored during a long-range re-orientation flight, and significantly longer flight durations and flight distances were observed. Thus, re-orienting bees explored a larger terrain than bees performing their first exploratory orientation flight. By displacing some bees after their first re-orientation flight, we could demonstrate that a single re-orientation flight seems to be sufficient to learn the new location of the hive. The flight patterns of re-orientation flights differed clearly from those of foraging flights. Thus, re-orientation flights represent a special exploratory behavior that is triggered by a change in the location of the hive.

## Introduction

Successful navigation of animals, including humans, depends on the degree of familiarity with the environment and the time lapse between learning and recall [1]. Exploration of the environment is a behavioral strategy used to build familiarity with a certain region that ensures a safe return to an important place (e.g. a nest site) or travel between places [2–5]. It is likely that exploratory behavior needs to be repeated when animals move (or are moved) to a new environment since the previously acquired information about the surroundings of the hive is no longer relevant. Nest-dwelling animals may have to switch nesting sites because of predation, parasitism, flood, fire, or food shortage. More specifically, absconding, migration and swarming are important processes in honeybee colonies during foraging and reproduction [6] with colony fission as a special strategy of colony multiplication that occurs in some species of social insects [7]. Honeybees move to a new nest location after swarming under natural conditions and experience a movement of the hive passively when beekeepers move colonies to another site. It has been shown that bees can learn the new location of the hive although problems might arise when the distance between the old and the new location of the hive is too short and other colonies exist near the old location of the hive [8–10]. Since honeybees usually re-nest within their original foraging range, they face the problem of adopting new responses to previously learned landmarks, and it has been shown that bees are indeed able to respond differently to previously learned landmarks after swarming [7,11]. Furthermore, bees are able to remember the old location of the hive after re-orientation to a new nest, indicating that the old memory is not lost or overwritten by the new memory. Maintaining a memory of the former nest site might be useful as a backup if the new colony fails, e.g. if the selected site proves to be undesirable or the queen dies before potential replacements have been raised. These observations are consistent with the idea that spatial memory is not necessarily organized in reference to a particular nesting site and is highly adaptive [7,11].

Becker [12] investigated the homing ability of experienced forager bees transferred into unfamiliar terrain. These experiments were later replicated with refined methods by Capaldi & Dyer [13]. Both studies tested animals in terrain outside of the foraging area and concentrated on a comparison of flights between young and experienced honeybees. The term ‘orientation flights’ was equally used for flights performed by young honeybees when they leave the hive for the first time and for the first flights of experienced foragers after the hive was transported to a new location. However, experienced foragers have already calibrated their sun compass as well as the visual odometer and collected information about the surrounding landscape. We therefore propose using different terms for these exploratory flights: flights of young honeybees that leave their nest for the first time are called ‘exploratory orientation flights’, while orientation flights of experienced foragers at the new location of the hive, either inside or outside of the original foraging range, are called ‘re-orientation flights’. Since Becker [12] and Capaldi & Dyer [13] did not monitor the flight trajectories of such re-orientation flights, only the duration of flights could be determined. Capaldi & Dyer [13] recorded a longer flight duration of re-orientation flights compared to orientation flights and hypothesized that re-orientation flights might cover more territory. Since exploratory orientation flights have been described in detail using the harmonic radar technique (honeybees: [14–16], bumble bees: [17]), the recording of re-orientation flights with the same technique makes it possible to test this hypothesis and to investigate whether exploratory behavior differs between these two situations. As described above, bees were shown to be able to reorient rapidly when the new location of the hive was within the original foraging range, as was the case in the present study. It was therefore interesting to also ask in which ways re-orientation flights differ from foraging flights to demonstrate the exploratory component of these flights. To address these questions, we used harmonic radar to record re-orientation flights of experienced foragers and compared the flight patterns and crucial flight parameters with exploratory orientation flights of young honeybees and foraging flights of experienced foragers.

## Materials and methods

### Experimental procedures

Two small observation hives (2000–3000 bees, *Apis mellifera*) were moved, at separate times, from their original locations to the same experimental field. The first hive was originally located in the village of Klein Lüben (Brandenburg, Germany) at the edge of a relatively small lawn (ca. 8500 m^2^), which was surrounded by trees and houses (52.965290° N, 11.858146° E). The new location (52.979187° N, 11.840356° E) in the experimental field was approximately 2 km linear distance away from the original location. The second hive was initially placed on a flat pasture close to a water channel, a cornfield and a creek (52.981261° N, 11.808448° E). The new location of hive 2 in the experimental field was approximately 2.1 km linear distance away from the original location on the pasture. It is likely that the new location in the experimental field, to which we transported the two hives successively, was within the original foraging range of both hives. The original location of hive 1 was chosen because of its differences to the experimental field since it was highly structured (houses, trees and bushes) and the original location of hive 2 because it was quite similar to the experimental field in both its low structured environment and the availability of natural food resources (see below). To ensure comparability of re-orientation flights recorded in the present study with exploratory orientation flights and foraging flights recorded in a previous study [15], all hives were positioned in the same experimental field and only flights recorded during the same time of the year were analyzed (see below). The experiments were carried out from 2011-08-25 to 2011-09-04 (hive 1) and from 2011-09-10 to 2011-09-13 (hive 2).

The bees were received from the local beekeeper and were allowed to forage for at least three weeks at their initial locations. At these locations, several foragers that returned with pollen to the hive were marked individually with number tags on the thorax one day before the hive was relocated to the experimental field. During transportation, the hive entrance was closed. The new location in the experimental area was surrounded by a flat, open pasture that featured several natural ground structures. These were different patches of grass and differently cut grasslands and we did not detect any obvious differences in these ground structures between 2010 (recording of exploratory orientation flights) and 2011 (recording of re-orientation flights). The experimental procedure did not differ between the two years either thus ensuring comparability between years. In both years, a transparent observation tube protruding 20 cm out of the hive was attached to the hive entrance to facilitate the registration of numbered bees exiting the hive. From 11 am to 5 pm, the hive entrance was observed closely by one or two experimenters. To ensure that all experimental bees’ flight activity was monitored, the hive entrance was closed every evening directly after the experiment. During the day, every numbered bee that left the tube was caught directly during take-off using a bottomless plastic water bottle, and a transponder was attached to the number tag on the thorax of the bee (see details below). When the bee returned, the transponder was removed and the bee went back into the hive until it was motivated to perform the next flight. Applying this procedure, we were able to record 32 re-orientation flights of bees that belonged to hive 1, and 16 re-orientation flights of bees that belonged to hive 2. Additionally, three bees of hive 1 were displaced approximately 200 m in two randomly chosen directions that were at least 120° away from each other (as seen from the hive) after their first re-orientation flight. The emphasis of these displacements was not detailed characterization of homing behavior but only testing whether the bees were able to find their way back to the hive quickly. When these bees returned from their first re-orientation flight or the first displacement, they were not allowed to enter the hive but instead were fed with sugar solution to ensure that they had enough energy to perform another flight. We transported the bees in a tube that was covered with a piece of dark cloth to the release site to ensure that they had no possibility to gain any visual information during displacement.

The method used to track flying bees with the harmonic radar (Raytheon Marine GmbH, Kiel, NSC 2525/7 XU) is described in detail by Menzel et al. [18]. The transponder had a weight of 10.5 mg and a length of 12 mm and was built by us following the procedure reported by Riley et al. [19]. Together with the number tag (Opalithplättchen, Bienen-Center Shop, http://www.bienencenter.com) the bee had to carry a total weight of 21 mg, which represents about 20 percent of the average weight of an unladen bee. There was no indication that the handling procedures or the attached transponder altered flight behavior since the flight duration of young honeybees that carried a transponder did not differ significantly from the flight duration of untouched bees or those that had undergone the same handling procedures but flew without a transponder [15].

The experimental field lacked distant landmarks, and the panorama of the horizon was flat within a 2° visual angle [18,20,21]. Because the angular resolution of the bee eye is considered to be defined by a visual angle of 2° [22,23], we assumed that the spatial modulation of the skyline did not serve as a guiding structure for bees (see discussion in Cheeseman et al. [24]; Cheung et al. [25]). The experimental field featured natural ground structures like different patches of grass or differently cut grasslands. An electronic weather station was positioned in the experimental field to record the temperature, humidity and wind speed. Furthermore, cloudiness (percentage of the sky covered with clouds in steps of 10) and visibility of the sun (no sun at all, sunshine) were estimated subjectively by one experimenter. Except for one flight of hive 2 where the sun was hardly visible at all, all recorded flights of bees from this hive were performed on sunny and relatively warm days with a low wind speed. For hive 1, flights performed under good as well as under unfavorable weather conditions (temperature < 20 °C and/or wind speed > 20 km/h and/or no sun at all) were recorded.

### Data analysis

For the comparison of re-orientation flights with orientation flights of young honeybees and foraging flights of experienced foragers that were recorded in the previous year [15], we analyzed only flights that were recorded during the same time period of the year (2010-08-25–2010-09-13) as the re-orientation flights (2011-08-25–2011-09-13). The handling procedure did not differ between these experiments. For hive 1, flight speed of bees that did not return to the hive from their first flight was compared with first long-range re-orientation flights that took the bees further than 30 m away from the hive using a *t*-test. Short-range re-orientation flights that were performed in the vicinity of the hive were not included because the bees often flew too low to be detected by the radar. For hive 2, flight speed of incomplete and complete flights could not be compared because of the low number of complete re-orientation flights. For the analysis of differences in flight duration and maximum range between exploratory orientation flights, re-orientation flights and foraging flights, we used an analysis of variance (ANOVA). The flight durations were log-transformed to comply with normal distribution and variance homogeneity. For post hoc comparison, we used the Tukey HSD test. Since the data for flight distance did not meet the requirements of the ANOVA, we used a Kruskal-Wallis test followed by Mann-Whitney *U* tests for post hoc comparisons. The Rayleigh test [26] was used to test for uniformity of the distribution of the directions of flight segments. To achieve this, each flight was dissected into segments: The first segment of a flight started with the first signal and ended at the second signal of this flight. The second segment of a flight started with the second signal and ended at the third signal of this flight and so on. Afterwards, the compass directions of each of these flight segments were determined. For the calculation of ANOVA, Kruskal-Wallis test, Mann-Whitney *U* test and *t*-test we used SPSS for Windows version 13.0. The Rayleigh test was carried out using a special package [27], and plots were generated with R version 2.15.1 [28].

### Ethical note

No licenses or permits were required for this research. The two bee colonies were provided by a local beekeeper and we returned the living colony at the end of the experiments. During experiments, there was no indication that the animals were restricted in their normal behavior.

## Results

### The pattern of re-orientation flights

The trajectories of re-orientation flights of bees belonging to the two hives from different original locations were recorded with harmonic radar in the same test area. However, not all bees returned to their hive. Such ‘incomplete flights’ as well as the recorded complete flights are summarized in Table 1. Most bees of hive 2 did not return to the hive from their first flight (81.3%). Although markedly more bees of hive 1 returned, the percentage of incomplete flights (40.6%) was still high. Five of these bees that did not return to the hive performed re-orientation flights beforehand. Interestingly, most incomplete flights recorded for bees of both hives were relatively straight and had their final signals close to the maximum range of the radar indicating that these bees flew beyond the detection radius of the radar (≈ 1 km). However, the flight speed did not differ significantly between first incomplete flights and first complete flights of bees that belonged to hive 1 (*t*-test, *t*(13) = 0.568, P = 0.580).

**Table 1:** Number of recorded incomplete and complete flights for the two different hives and the classification of complete flights in short-range and long-range flights.

Hive	Recorded flights	Incomplete flights		Complete flights		Complete flights			
						Short-range		Long-range	
	N	N	%	N	%	N	%	N	%
1	32	13	40.6	19	59.4	9	47.4	10	52.6
2	16	13	81.3	3	18.7	0	0	3	100

**Fig 1:**
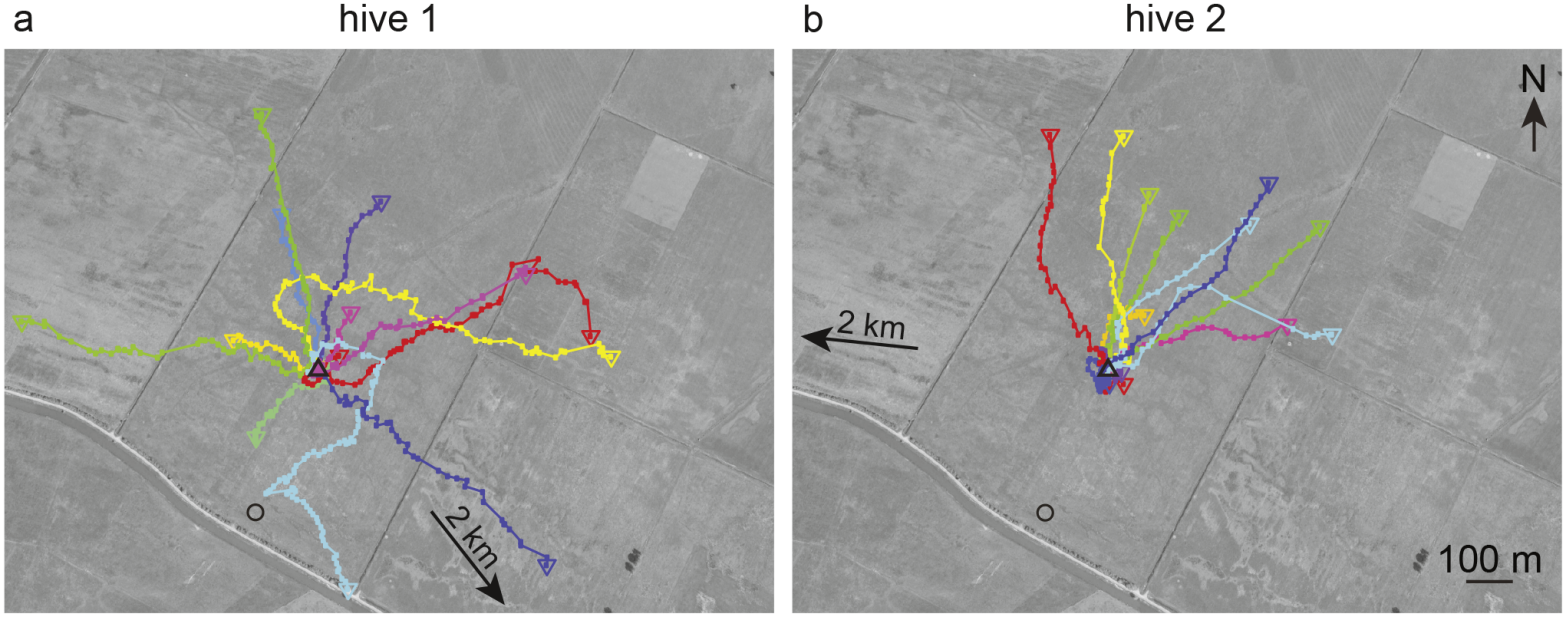
Flight trajectories of bees that did not return to the hive. The flight trajectories of all incomplete flights of bees that belonged to hive 1 (a, N = 13) and of those that belonged to hive 2 (b, N = 13) are shown in a different color for each bee. The last positions of the bees tracked by the radar are marked by the colored triangles. The black triangle marks the location of the hive and the circle marks the location of the radar. The arrows next to the hives indicate the direction and distance to the original location of the hive. The filled squares on flight paths are the positions given by radar (usually every three seconds).

The compass directions of these final signals differed between the two hives. While the bees of hive 1 directed their flights in various directions (Fig 1a), all bees of hive 2 left the radar range north or northeast (Fig 1b). The direction of the beeline connecting the old and the new location of the hive was southeast for hive 1, which was indeed chosen by one bee, and west for hive 2, a direction not chosen by any bee of this hive.

**Fig 2:**
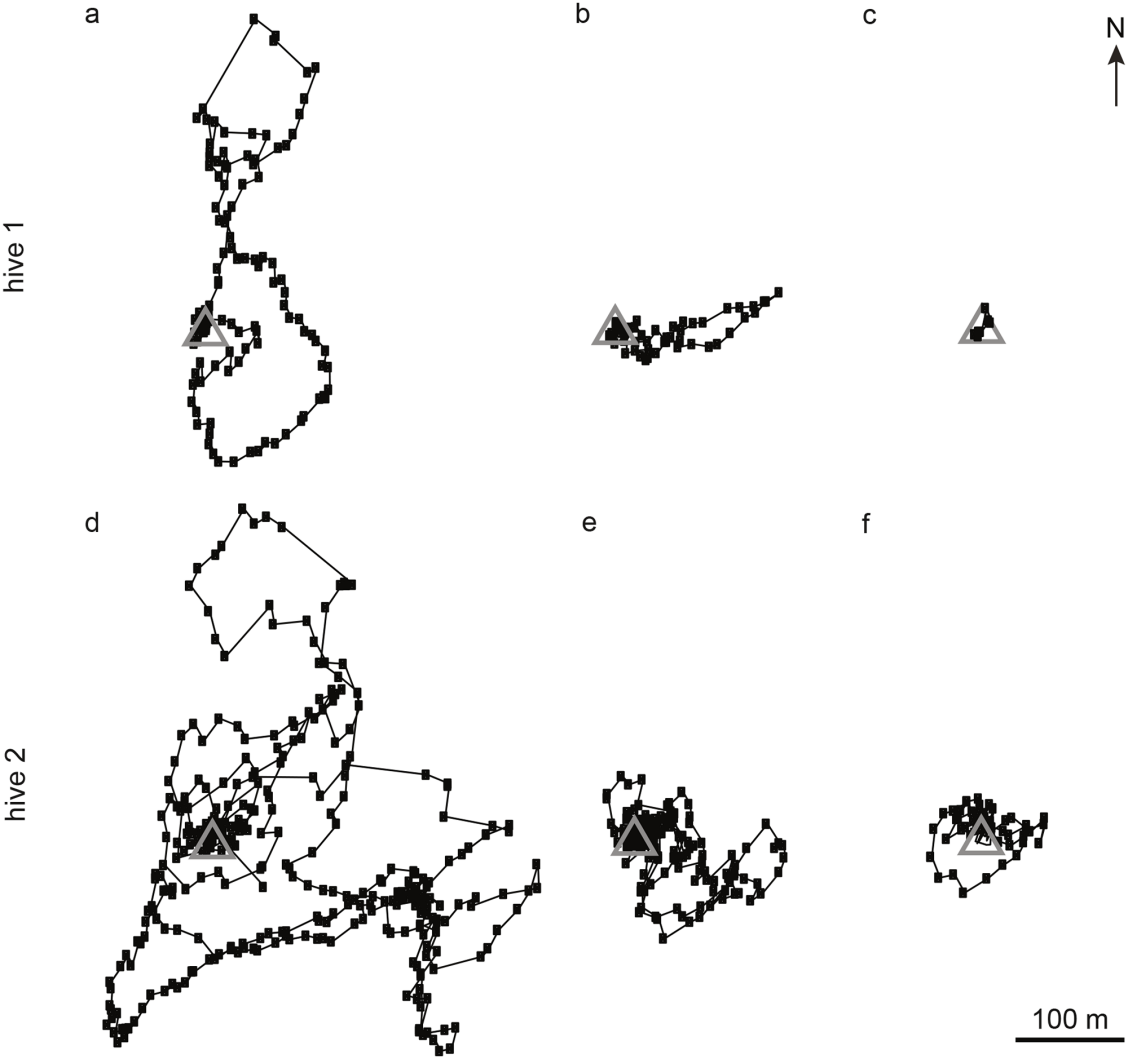
Representative examples of flight trajectories of re-orientation flights. For hive 1 (a–c) one exemplary flight trajectory for each flight pattern is shown: (a) long-range flight of a bee that explored more than one sector of the surrounding landscape, (b) long-range flight of a bee that explored one sector of the surrounding landscape only, (c) a short-range flight (see text for details). The other recorded complete flights of this hive are presented in S1 and S2 Figs. For hive 2 (d–f), all recorded complete flights are shown. The gray triangle marks the location of the hive and the filled squares on flight paths are the positions given by radar (usually every three seconds).

For hive 1, 14 complete first re-orientation flights were recorded. Seven of these flights took the bee more than 30 m from the hive (long-range flights, flight example: Fig 2a). Five of these seven bees explored the landscape in more than one direction (flight example: Fig 2a), the other two explored only one sector of the surrounding landscape (flight example: Fig 2b). Exploration of only one sector is typical for exploratory orientation flights of young honeybees [14,15]. We speak of a sector because the outbound and inbound components of these flight trajectories are close to each other. The flight pattern of the re-orientation flight shown in Fig 2b meets this criterion but since in total only 3 out of 13 bees explored only one sector of the surrounding landscape during a long-range flight (see also the description of multiple re-orientation flights of single bees of hive 1 and re-orientation flights of hive 2 below) this behavior seemed to be the exception in reorienting bees. While only two of the seven long-range re-orientation flights were performed under unfavorable weather conditions (temperature < 20 °C and/or wind speed > 20 km/h and/or no sun at all), six out of seven flights that brought the bee less than 30 m away from the hive (short-range flights, flight example: Fig 2c) were performed under such weather conditions. All flights that are not shown in Fig 2 are presented in S1 and S2 Figs. S2 Fig shows multiple flights of single bees, but none of these bees explored the further surroundings of the hive during more than one flight. Therefore, the spatial organization of consecutive flights could not be analyzed.

For hive 2, only three complete first re-orientation flights were recorded, because all other 14 bees of this hive did not return to the hive from their first flight (see above and Fig 1b). These three bees performed flights that brought them further than 30 m away from the hive and explored more than one sector of the surrounding landscape (Fig 2d–2f).

Next, we asked whether the landscape structure of the experimental field influenced the flight pattern of first re-orientation flights. For bees from hive 1, only flights that took the bee more than 30 m from the hive were analyzed, since flights that were performed in the vicinity of the hive consisted of an accumulation of only a few radar signals close to each other with no clear direction of the flight trajectory (see Fig 2c). We hypothesized that extended landmarks on the ground might have influenced the flight directions. Therefore, flight trajectories were dissected into single flight segments (see Methods) and we calculated the frequencies of their directions. The analysis revealed that the directions of flight segments were distributed uniformly for flights of hive 1 (Rayleigh test: *P* = 0.065, number of flight segments = 1038) as well as for flights of hive 2 (Rayleigh test: *P* = 0.356, number of flight segments = 613).

### Comparison of flight parameters

In a previous study [15], exploratory orientation flights of young honeybees and foraging flights of experienced foragers were recorded with harmonic radar in the same experimental field as in this study. We are aware that a comparison between data collected in two different years from different colonies might be considered problematic because ideally such a comparison should be based on parallel studies performed at the same time, in the same environment and with animals from the same colony. It was not possible to achieve these conditions with the limited work force of our group and the constraints associated with the harmonic radar. We, therefore, aimed for a compromise by performing the experiments at the same time of the year in the same experimental field. Fortunately, the ecological and weather conditions in the test area were similar in the two years in question.

**Fig 3:**
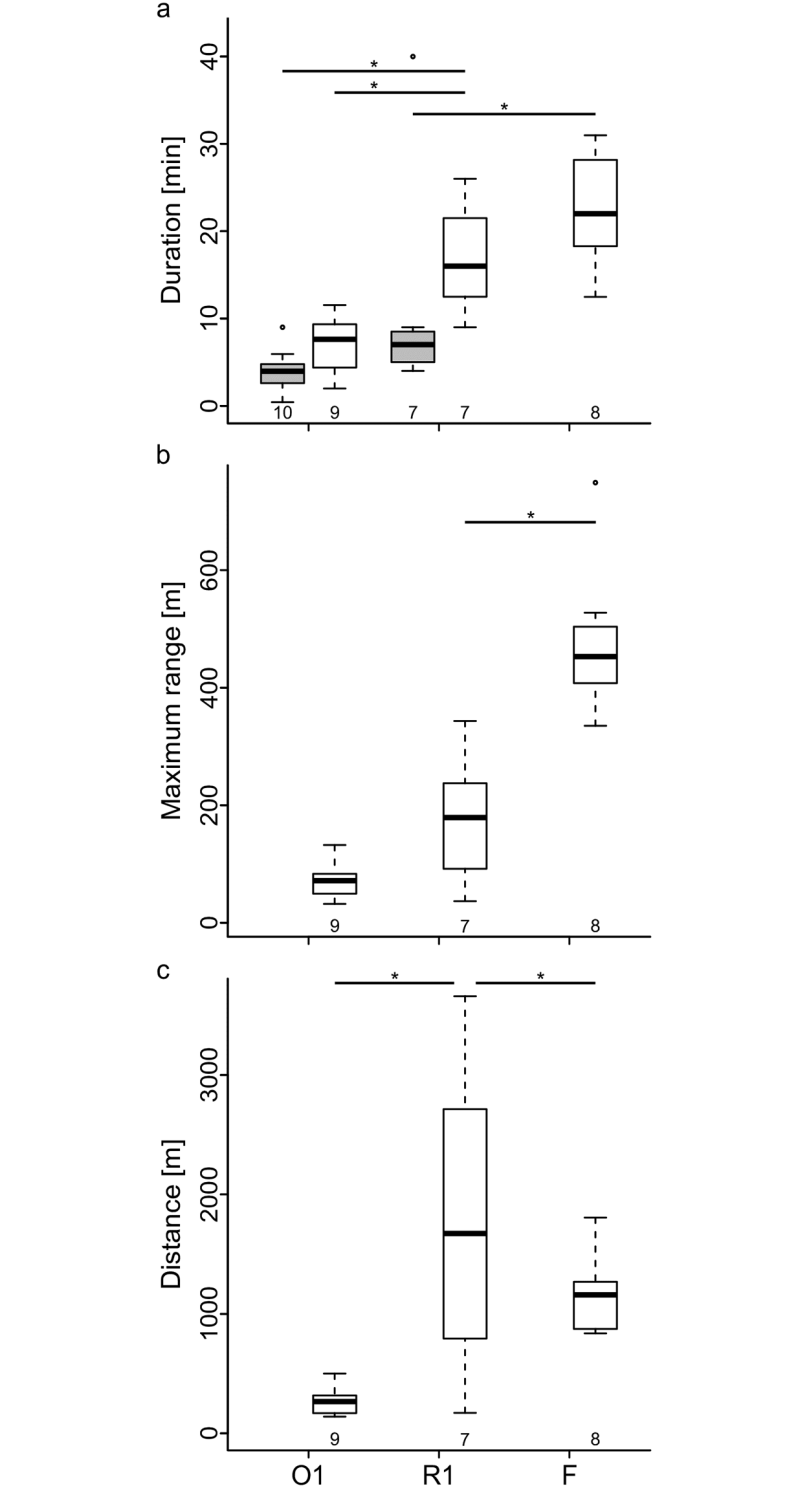
Comparison of flight parameters between first exploratory orientation flights, re-orientation flights, and foraging flights. (a) Flight duration of short-range flights (gray) and long-range flights (white). (b) Maximum range of long-range flights. (c) Total distance travelled during long-range flights. Only data from hive 1 is shown as the data from hive 2 was insufficient for quantitative analysis. O1 = First exploratory orientation flight, R1 = First re-orientation flight, F = Foraging flight. Box plots show the median (black line), the interquartile range (box), the minimum and maximum value within 1.5 times the interquartile range of the box (whiskers) and the outliers (circles). The number underneath each box plot gives the sample size. Statistics: Analysis of variance (ANOVA, a and b), Kruskal-Wallis test (c). Significant differences (post-hoc test *P* < 0.05) are marked by *.

To determine differences between these two types of flights and re-orientation flights, we compared the characteristic parameters of flights that were performed during the same time period of the year (see Methods). In the previous study, exploratory orientation flights were classified into short- and long-range orientation flights based on the maximum range (≤30 m; >30 m, respectively) and the re-orientation flights recorded in the present study were classified in the same way. One characteristic flight parameter is flight duration and Fig 3a shows those of first exploratory orientation flights, first re-orientation flights (hive 1), and foraging flights. A comparison of all groups revealed significant differences between groups (ANOVA: F_4,36_ = 11.390, p < 0.001). A post hoc Tukey HSD test showed that the duration of first re-orientation flights of bees that flew further than 30 m away from the hive had a significantly higher duration than short-range and long-range orientation flights (p < 0.05, Fig 3a) but did not differ significantly from foraging flights. Short-range re-orientation flights had significantly lower flight durations than foraging flights (p < 0.05). Since short-range flights have a maximum range of less than 30 m by definition and consist of only a few radar signals close to the hive, flight duration is the crucial parameter for the comparison of short-range re-orientation and orientation flights.

Long-range flights may vary in both maximum range and total distance flown. A major advantage of the experimental field was its lack of natural food sources near the hive. Since all foraging areas were located more than 270 m away from the hive, flights with a maximum range below 270 m were likely not to be foraging flights. The maximum range of re-orientation flights was significantly lower than those of foraging flights (ANOVA: F_2,21_: 38.850, p < 0.001; Post hoc: Tukey HSD test, p < 0.001; Fig 3b). However, one re-orientation flight had a maximum range of 343 m, but this particular bee travelled a total distance of 3660 m—a distance much longer than those of typical foraging flights. In general, the total flight distance differed significantly between groups (Kruskal-Wallis test: H_2_ = 12.223, p = 0.002). Post hoc comparisons showed a significantly longer flight distance of re-orientation flights than orientation flights (Mann-Whitney *U*-test: *U*(9,7) = 9, p = 0.016, Fig 3c). Thus, long-range re-orientation flights can be differentiated from foraging flights based on the lower maximum range and from orientation flights based on the longer total flight distance and flight duration.

### Displacements of bees after one re-orientation flight

**Fig 4:**
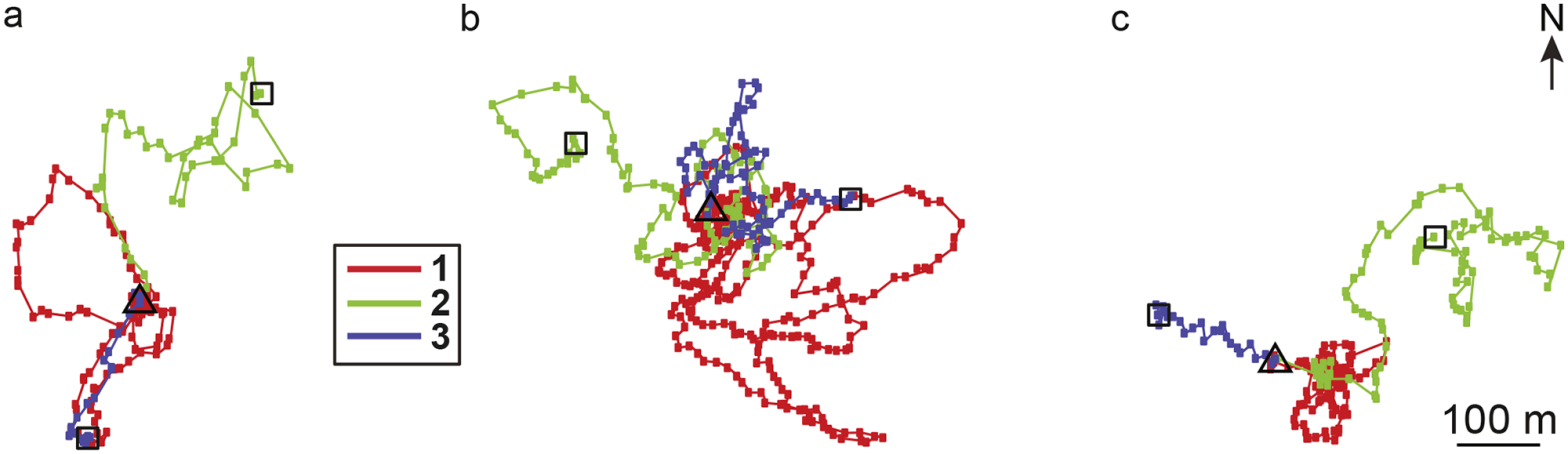
Flight trajectories of bees that were displaced after their first re-orientation flight. All bees were from hive 1 and were released successively at two sites that were in different directions from the hive. The first flight (red) shows the first re-orientation flight, the second (green) and third (blue) flights are the ones after displacement. The inset displays for all figures the color coding of the sequence of flights. The release sites after displacement are marked by squares, and the filled squares on flight paths are the positions given by radar (usually every three seconds). The location of the hive is marked by the triangle.

To investigate if bees were able to return quickly to the hive after one re-orientation flight, three bees of hive 1 were displaced two times ≈ 200 m away from the hive in different directions (Fig 4). All these bees had performed a re-orientation flight that brought them further than 30 m away from their hive with similar flight durations (15, 16 and 17 min) before the displacements. After a displacement, all bees were able to return quickly to the hive (mean ± SD: 4.8 ± 1.8 min, range: 3–7 min). Both curvy flights (e.g. Fig 4a, green flight trajectory) and relatively straight flights back to the hive (e.g. Fig 4a, blue flight trajectory) occurred.

## Discussion

When a whole colony is transported to a new site, foraging bees learn the location of the hive in the novel landscape during an exploratory phase of re-orientation. Foraging bees performing such re-orientation flights have already calibrated their sun compass and the visual odometer, because they had extensive experience of foraging at the old location. We therefore expected that they would focus their re-orientation flights on learning about the new landscape structure. Therefore, the pattern of re-orientation flights might well differ from those of initial exploratory orientation flights of young bees. Since it was unclear how far the exploratory component of the re-orientation flights ranged and whether bees foraged during their first re-orientation flight, we also compared these flights with foraging flights of experienced foragers. We used the harmonic radar to capture the flight patterns of re-orientation flights and monitored bees from two different hives that were positioned at different locations before they were transported to the same experimental field.

Hive 1 was located in a small village on a lawn most likely exposed to richer forage around the hive than in the surrounding agricultural area, while hive 2 was located on a flat pasture with a low abundance of profitable forage rather similar to the conditions in the experimental field. We chose these original locations to investigate if the flight pattern of re-orientation flights differs depending on the structure of the learned landscape. Unfortunately, nearly all bees that belonged to hive 2 did not return to their hive from their first flight, making such a comparison impossible. The flight speed of these incomplete flights did not differ significantly from that of complete long-range re-orientation flights of bees that belonged to hive 1. The most likely explanation for the unusually high percentage of incomplete flights is that these bees headed off to the feeding sites they had visited before without noticing that their hive had been moved to a new location. This could also explain the differences in the range of directions since the village featured profitable food resources in all directions (hive 1) while the landscape that surrounded the original location of hive 2 did not. Most of the bees that did not return to the hive left the detection radius of the radar: the search flight [21,29] that must have followed at the end of the flight towards a memorized food source was thus not recorded. Since the two hives were both located approximately 2 km away from the experimental field, it is likely that they were displaced within the original foraging range. The exact foraging radius of a honeybee colony is known to depend on the abundance of profitable forage, but distances up to 6 km from the nest may be reached [30–32]. Thus, bees that did not return to the hive most likely flew back to the old location of their hives. However, this remains a hypothesis that needs to be tested as we did not monitor their arrival at the old locations.

Bees that noticed that their hive was moved performed re-orientation flights to gather information about the new location. The flight pattern of complete re-orientation flights varied in at least two ways: (1) range—we recorded both short-range flights that stayed close to the hive as well as flights that ranged further; (2) number of directions taken from the hive during a single flight. The exploration of only one sector of the surrounding environment is typical for orientation flights of young honeybees and characterized by outbound and inbound components that are located close to each other. Since all bees except for two visited more than one sector during a flight, the exploration of various directions seems to be the typical pattern of re-orientation flights. Thus, re-orientation flights show differences but also similarities with exploratory orientation flights of young bees: Just as exploratory orientation flights of young honeybees, re-orientation flights can be classified into short- and long-range flights with short-range re-orientation flights occurring more frequently under unfavorable weather conditions. Yet unlike exploratory orientation flights, re-orienting bees usually explored the new landscape in more than one direction, which is reflected in a longer duration and further distance of long-range flights. Longer lasting re-orientation flights compared to orientation flights were also found by Capaldi & Dyer [13] and we can confirm their hypothesis that this difference is due to the larger amount of terrain explored during a re-orientation flight with the present results. They did not differentiate between short- and long-range flights, but since they only performed their experiment on warm and sunny days, it is likely that their test bees only performed long-range flights. Taken together, short-range re-orientation flights of experienced foragers that performed re-orientation flights after their hive was transported to a new location were comparable to short-range orientation flights of young bees that leave their hive for the first time while long-range re-orientation flights differed in flight pattern, flight duration and flight distance travelled.

Bees can perform two more typical sorts of flights: foraging flights and scout flights. Our data show that re-orientation flights can be differentiated from foraging flights based on the lower maximum range, which means that no natural food resources were reached because the experimental field featured those only further away, as well as the flight pattern, since foraging flights to an established food resource typically follow an almost straight line from the hive to the resource [33]. Thus, the flights recorded in the present study focus entirely on the exploration of the new terrain. Although the flight pattern and flight parameters of scout flights have not been investigated in detail so far, it is likely that these are long-lasting and extensive flights like the one recorded in a previous study [15]. Thus, besides flight duration and distance, the area explored during a flight presumably is a critical parameter for the distinction of re-orientation flights and scout flights.

It has been shown that honeybees can rapidly learn the new location of their hive [7,12]. Capaldi and Dyer [13] were able to demonstrate that one re-orientation flight can be sufficient for successful homing after a displacement. To investigate if bees of the present study were also able to return quickly to the hive after a displacement, we displaced bees of hive 1 that performed only one long-range re-orientation flight two times in different directions. After displacement, all these bees were able to return quickly to their hive. Although the sample of homing flights was rather small, these results suggest that for experienced foragers even a single re-orientation flight is sufficient to learn the new location of a displaced hive. A continuation of flights after reaching the hive was not observed for young honeybees displaced after one exploratory orientation flight [16]. Thus, the higher experience of foragers seems to facilitate further exploratory behavior. This higher experience is likely to include trained motor performance and calibration of the visual odometer as well as the time-compensated sun compass.

Taken together, we showed that the exploratory behavior of re-orienting bees is more wide ranging compared to young, inexperienced bees. Honeybees adapt to changing weather conditions by choosing between short-range and long-range re-orientation flights, and one long-range re-orientation flight seems sufficient for learning the new location of the hive.

## Supporting information

S1 Fig Trajectories of re-orientation flights of bees that belonged to hive 1. The figure shows all flights of bees for which only one flight was recorded that are not included in the figures of the main text. In (a) the last signal at the hive is missing. The hive is marked by the triangle.
(TIF)

S2 Fig Examples of flight trajectories of bees with more than one recorded re-orientation flight. All four bees belonged to hive 1. Re-orientation flights that took the bees not further than 30 m away from the hive (a: flights 2 and 3; b: flight 1, c: flight 1, d: flight 1) are hard to see in this figure since they consist of only a few radar signals close to the hive. The black triangle marks the location of the hive. The inset displays the color coding of the sequence of flights bees performed after they left the hive.
(TIF)

S1 Table Data set flight parameters. (XLSX)

S2 Table Data set flight segments. (XLSX)

S1 Textfiles Textfiles of all flights. The duration of a flight was measured at the hive. Bees usually flew low at the start and end of their flight and could not be detected by the radar during this time. Therefore, the time between the first and the last signal in the textfile deviates from the flight duration recorded at the hive by monitoring take off and landing of the bees. For incomplete flights the time between the first and the last signal was used to calculate the flight duration, but these values were not analyzed any further. Due to the usage of different watches to record the start- and end time of a flight there might be deviations from the times in the textfile because not all watches were synchronized with the radar.
(ZIP)
