# Supplementary figures and images for: Exploratory behavior of re-orienting foragers differs from other flight patterns of honeybees
Source: PLoS One. 2018 Aug 29;13(8):e0202171. doi: 10.1371/journal.pone.0202171 (PMC6114720; doi:10.1371/journal.pone.0202171)

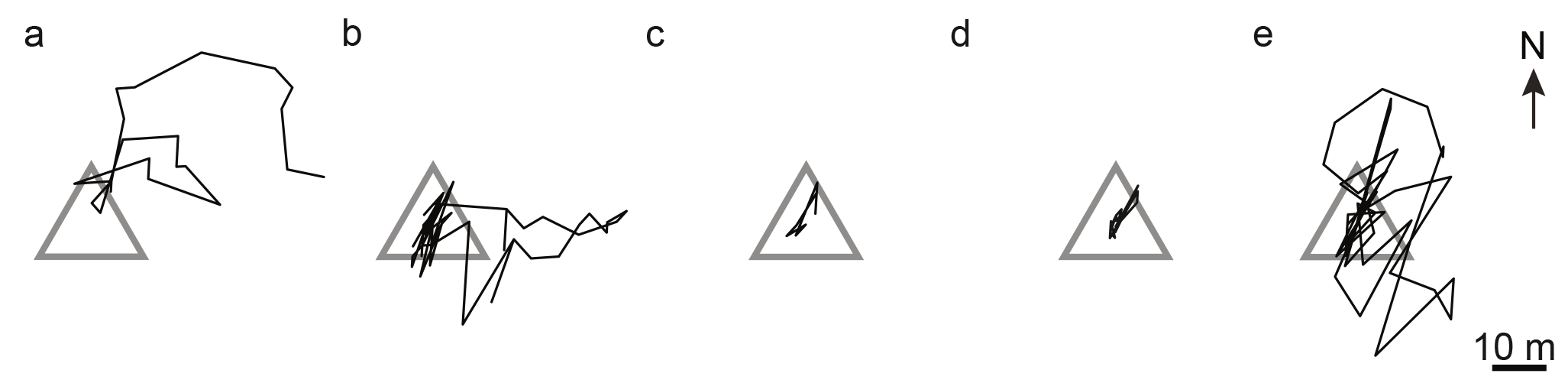

Supplement: S1 Fig — The figure shows all flights of bees for which only one flight was recorded that are not included in the figures of the main text. In (a) the last signal at the hive is missing. The hive is marked by the triangle. (TIF) [file pone.0202171.s001.tif]

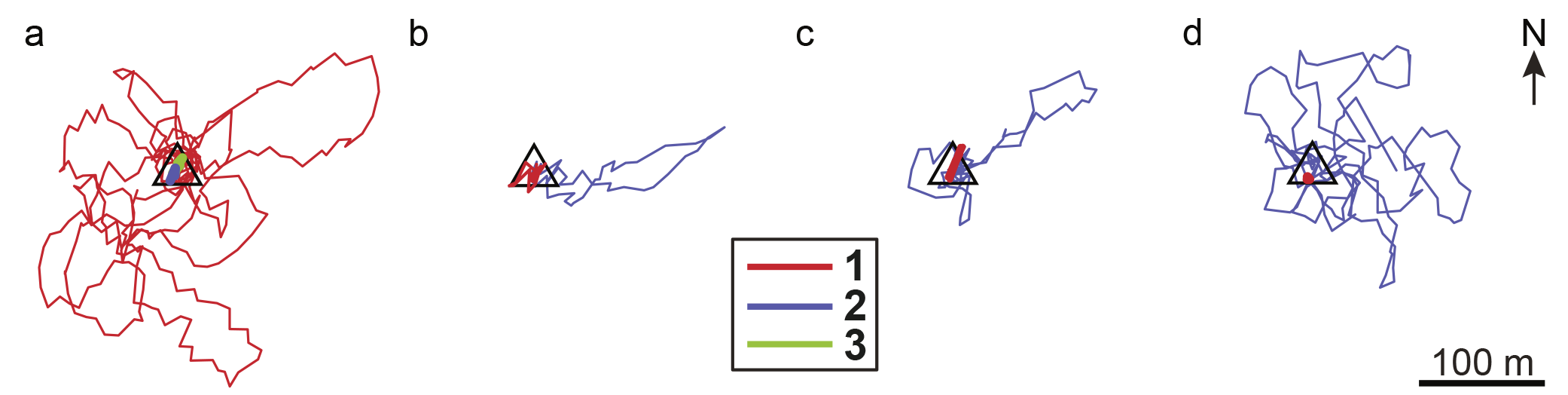

Supplement: S2 Fig — All four bees belonged to hive 1. Re-orientation flights that took the bees not further than 30 m away from the hive (a: flights 2 and 3; b: flight 1, c: flight 1, d: flight 1) are hard to see in this figure since they consist of only a few radar signals close to the hive. The black triangle marks the location of the hive. The inset displays the color coding of the sequence of flights bees performed after they left the hive. (TIF) [file pone.0202171.s002.tif]
